# Exploring the key microbial changes in the rhizosphere that affect the occurrence of tobacco root-knot nematodes

**DOI:** 10.1186/s13568-020-01006-6

**Published:** 2020-04-15

**Authors:** Kuo Huang, Qipeng Jiang, Liehua Liu, Shuting Zhang, Chaoli Liu, Haitao Chen, Wei Ding, Yongqiang Zhang

**Affiliations:** 1 College of Plant Protection, Southwest University, Chongqing, 400715 China; 2 Chongqing Institute of Tobacco Science, Chongqing, 400715 People’s Republic of China

**Keywords:** Root-knot nematode, Soil-borne disease, Microbial community, Pseudomonas

## Abstract

Root-knot nematode (RKN) disease is a soil-borne disease. However, most studies on RKN have focused on the screening of agents and the cultivation of resistant varieties, and reports on the interaction of RKNs with soil microorganisms are few. In this study, we performed Illumina high-throughput sequencing to analyze diseased and healthy soil and the microbial-community changes in rhizosphere soil after microbial treatment (*Pseudomonas flurescens*, *Bacillus subtilis*, *Paecolomyces lilacinus*). Results showed significant differences in the bacterial community richness and diversity between diseased and healthy soil and the presence of different microbial species. After treatment, the richness and diversity of microbial communities in soil, as well as the number and incidence of second-stage juvenile of RKNs, decreased. Through linear discriminant analysis effect size, Pearson correlation, and Venn diagram analysis, we screened five genera that were closely related to disease occurrence, among which *Pseudomonas* was most related to disease inhibition. Our results suggested that the occurrence of tobacco RKN was related to changes in soil microbial communities, and that the interactions among *Pseudomonas*, *Bryobacter*, *Variibacter*, *Coniochaeta*, and *Metarhizium* affected the health of rhizosphere soil.

## Introduction

Root-knot nematode (RKN) (*Meloidogyne* spp.) infestation is a soil-borne disease that has a wide-ranging and serious damage worldwide; it is difficult to control and its incidence has continuously increased in recent years (Kerry 2000; Jeger et al. 2018). According to Food and Agriculture Organization of the United Nations (FAO) statistics, the annual loss caused by plant parasitic nematodes is more than 157 billion dollars worldwide; hence, infestation with such nematodes has been considered the fourth type of invasive disease that harms plants (Abad et al. 2008; Oka et al. 2000; John et al. 2013). Tobacco RKN disease occurs in the major tobacco areas in China, and the yield loss can reach 30% to 50%, and it can also induce compound infection diseases, such as bacterial wilt and black shank (Qiu 2010; Li et al. 2013). Resistant varieties and non-host plants are difficult to promote; hence, the current production is still based on chemical agents (Williamson and Hussey 1996). The use of chemical nematicides may be harmful to non-target organisms, and can pollute the environment (Chitwood 2002). Therefore, regulating the occurrence of diseases by finding key microbial changes has become the key to solving soil-borne diseases (Sharon et al. 2001).

Soil-borne diseases are caused by the imbalance in soil ecosystems, such as changes in planting conditions, changes in climate and environment, and changes in treatment measures; hence, understanding the mutual equilibrium relationship between the original soil microorganisms, and balance of soil ecosystems is the key to prevent the disease from continuing to occur and keep the healthy growth of crops (Liu et al. 2016). Microbial imbalances in the “plant-soil-microorganism” ecosystem play an important role in the occurrence of diseases (Classen et al. 2016). Some soil-borne pathogens, *Rhizoctonia solani*, *Ralstonia solanacearum*, *Fusarium oxysporum*, and so on, have recently been confirmed to be closely related to soil microecological imbalance (Navarrete et al. 2013). Around the root system, soil microbes interact with pathogens, and the balance is destroyed, causing disease to occur (Nobori et al. 2018). RKN causes microecological changes in tobacco; however, its interaction with rhizosphere microorganisms has rarely been studied.

The rhizosphere is a tiny 1–2 mm area outside the root of plants (Philippot et al. 2013). It is surrounded by soil and contains a large number of microorganisms that may be beneficial, harmful, and neutral to plant growth; the first two can interact to affect the growth and health of the plants (Zhang et al. 2017a, b). Some studies have shown that balance between rhizosphere microbes is one of the important reasons for pathogen outbreaks and the rhizosphere environment from health to disease (Zobiole et al. 2011). Development and utilization of biocontrol agents are considered to be one of the most promising approaches for controlling soil-borne diseases (Kerry 2000). Addition of biocontrol agents to the soil can directly or indirectly affect the rhizosphere microorganisms and regulate the occurrence of crop diseases (Xie et al. 2019). Traditional research on the biological control of plant diseases primarily focuses on the interaction between plants, pathogens, and biocontrol bacteria but ignores the role of microbial groups (Yang et al. 2018a, b). Rhizosphere microorganisms are directly involved in the defense mechanisms of plant diseases (Mendes et al. 2013). The most effective approach to prevent and control RKN disease is to regulate the rhizosphere microbial community structure and maintain soil health.

Recent studies have shown that a small class of key groups are present in the rhizosphere microbial community, and they play a leading role in promoting disease occurrence (Berg and Smalla 2009; Liu et al. 2016; Berendsen et al. 2012; Lawson et al. 2019). Therefore, we extracted 16S rRNA and 18S rRNA genes from the total DNA of perennial diseased and perennial healthy tobacco rhizosphere soils to further analyze the rhizosphere microbial composition under natural conditions. Subsequently, we analyzed and verified the microbial differences between the two by pot and field experiments of the biological control to select the key microbial groups that may affect the occurrence of tobacco RKN disease. This study aims to identify key microorganisms that indicate the occurrence of tobacco RKN disease and provide theoretical support for disease monitoring and regulation in agricultural production.

## Materials and methods

### Site description and sample collection

The samples were collected in Huili County (26°49′44′ N and 102°16′56′ E) Sichuan, China. This area is a representative area for the occurrence of RKN. We collected rhizosphere-soil samples from three diseased-soil (D) and one healthy-soil (H) sites during tobacco maturity (100 days after transplanting). The sites were not more than 1 km apart, and tobacco continuously grows in them every year. Five seedlings of tobaccos were randomly selected from each plot using a five-point sampling method. The complete root system was dug out, the loose soil particles were removed from the large clods and roots, and the soils attached to the roots were gently swept with a brush. The soil was passed through a 2 mm mesh screen to remove debris and placed in a 1000 mL sterile plastic bag. The mixture was manually mixed in triplicate and immediately transported to the Soil Analysis Laboratory of Southwest University of China and stored at ambient temperature for 24 h (2–4 °C). The basic physical and chemical properties of each soil sample are detailed in Additional file 1: Table S1.

### Potted and field experiments

The soil samples around the tobacco roots were collected from the three RKN sites and mixed for greenhouse pot experiment. The biocontrol agents used in the experiment, namely, *Bacillus subtilis* (ACCC:04179), *Pseudomonas fluorescens* (ACCC:10040), and *Paecilomyces lilacin* (ACCC:30678), were purchased from the Agricultural Culture Collection of China. A 6-week-old tobacco variety Honghua Dajinyuan was transplanted into soil samples and colonized for 2 days, and then each treatment was inoculated with 5 mL of *Bacillus subtilis*, *Pseudomonas fluorescens*, and *Paecilomyces lilacinus* suspension (3.8 × 10^7^ CFU/mL), labeled as Bs, Pf, and Pl, respectively. The untreated diseased soil was labeled as P_D. Subsequently, the inoculated tobacco was placed in an incubator at 28 °C under a light/dark cycle for 14/10 h. Each treatment was repeated three times with five tobaccos per replicate. A total of 100 g of rhizosphere soil was collected 90 days after the treatment, and the population of the second-stage juveniles of RKN (J2) was determined after separation (The J2 period is the key period when the root-knot nematode has the characteristics of infecting plant roots, so as to indicate the degree of harm.) (Sharon et al. 2001). At the same time, rhizosphere soil was collected for subsequent studies according to the method stated in section “*Site description and sample collection”*.

The field trial was conducted at the sample-collection site on April 18, 2018 when the tobacco seedlings were transplanted. The test design has four treatments: (1) CK, without any treatment; (2) F_Bs, 100 billion live spores/g *Bacillus subtilis* 1.5 kg/hm^2^ irrigation; (3) F_Pf, 300 billion live spores/g *Pseudomonas fluorescens* 0.5 kg/hm^2^ irrigation; and (4) F_Pl, 5 billion live spores/g *Paecilomyces lilacin* 30 kg/hm^2^ irrigation. The microbial agents used in all field trials were purchased from Jining Yuyuan Biotechnology Co., Ltd. The experiment was carried out by random cell processing, and each treatment was repeated three times. Each cell area was 66.7 m^2^, 110 tobacco plants were planted, and protection was set around. The remaining agronomic measures for each treatment are the same. Ninety days after processing, the incidence rate of RKN disease was calculated.

### DNA extraction, PCR amplification, and sequencing

Total genomic DNA was extracted from 0.5 g of soil from each sample using the FastDNA™ SPIN Kit for Soil (MP Biomedicals, Illkirch, France) according to the standard protocol. Genomic DNA purity and concentration were measured using a ThermoFisher Scientific (MULTISKAN GO, USA) microplate reader. Primers were designed as follows: 515 forward (5′-GTGCCAGCMGCCGCGG-3′) and 806 reverse (5′-GGACTACHVGGGTWTCTAAT-3′) were used to amplify the V4 region of the 16S rDNA gene; and SSU0817 forward (5′-TTAGCATGGAATAATRRAATAGGA-3′) and 1196 reverse (5′-TCTGGACCTGGTGAGTTTCC-3′) were used to amplify the V5–V7 region of the 18S rDNA gene (Zhang et al. 2017a, b; Xiong et al. 2017).

The PCR parameters were as follows: an initial denaturation at 95 °C for 3 min, followed by 28 cycles of denaturation at 95 °C for 30 s, annealing at 55 °C for 30 s, elongation at 72 °C for 45 s, and a final extension at 72 °C for 10 min. PCR was performed in triplicate using 20 µL of reaction solutions containing the following: 2.0 µL of 10 × buffer, 2.0 µL of dNTP (2.5 mM), 0.2 µL of rTaq Polymerase (Takara), 0.2 µL of BSA, 0.8 µL of each of forward and reverse primers, and 10 ng of template. Amplified products were ran on a 2% agarose gel for identification, and the samples with bright main bands between 400 and 450 bp were selected for further experiments. Amplicon was combined at approximately equal amplification intensity ratios, purified using an AxyPrep DNA Gel Recovery Kit, and submitted to the next-generation sequencing laboratory at Majorbio Biopharm Technology Co., Ltd. (Shanghai, China) for Illumina paired-end library preparation, cluster generation, and 250-bp paired-end sequencing. Sequences were deposited into the NCBI short-reads archive database under Accession Number SRP226740.

### Bioinformatics and statistical analyses

Splicing and filtering were performed on raw data to obtain valid data and ensure the accuracy and reliability of the results. Raw Illumina fastq files were demultiplexed, quality filtered, and analyzed using QIIME v1.7.0 (Caporaso et al. 2010). Operational taxonomic units (OTUs) were selected with 97% similarity. The following criterion was used to identify high-confidence OTUs: any sample with a collective abundance of more than 20 reads. Subsampling was performed using the minimum length of the sample sequence. The overall microbial distribution in the different samples was compared and tested to demonstrate that calculation was made with OTU based on the taxonom using Origin 9.0 software for histogram analysis.

The Chao and Shannon indices were calculated to measure the α-diversity and determine the species richness in each sample. At the OTU level, hierarchical clustering analysis and principal co-ordinates analysis (PCoA) of each sample were performed to determine the β-diversity of the sample and the difference in microbial species.

Linear discriminant analysis (LDA) effect size (LEfSe) was shown to identify taxa with significant differential abundances between samples. The factorial Kruskal–Wallis sum–rank test (α = 0.05) was used in LEfSe analysis, which was followed by LDA to estimate the effect size of each differentially abundant feature (logarithmic LDA score > 2.0) (Segata et al. 2011). Significant taxa were used to generate taxonomic cladograms, which illustrated the differences between sample classes on the website http://huttenhower.sph.harvard.edu/galaxy. Furthermore, in the case of decentralized redundant data, the level of classification is limited by the domain and the genus. After comparing the diseased soil and the healthy soil, the group with significant difference was found at the genus level, and then the Venn diagram was compared with the correlation between J2 and bacterial microorganism (significant difference was observed in the relative abundances of D and H) to determine similar groups.

Linear regression analysis (Pearson correlation) was performed to evaluate the relationships between the microbial community and the population of the second-stage juvenile of RKN. For each group of data, the mean and standard error were calculated by ANOVA and Turkey’s honest significant difference test (P < 0.05) using SPSS 19.0 (SPSS Inc., Chicago, IL, USA).

## Results

### Comparison of the microbial differences between diseased soil (D) and healthy soil (H)

Through high-throughput sequencing analyses of 16S rDNA and 18S rDNA, 346246 valid bacterial sequences and 179,952 effective fungal sequences were read from 12 field samples and divided into 5942 bacterial OTUs and 238 fungal OTUs.

**Fig. 1:**
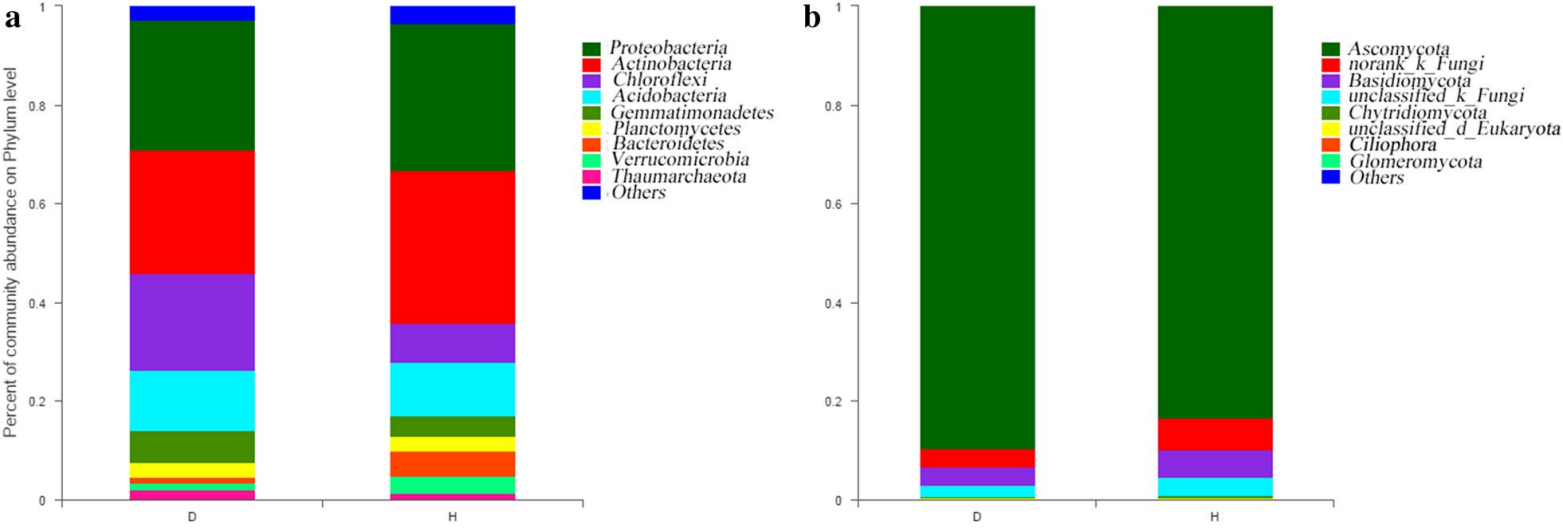
Bacterial (**a**) and fungal (**b**) community composition at the level of the diseased soil (D) and healthy soil (H)

A total of 5942 bacterial OTUs were assigned to 42 different phyla: D contained 39 and H contained 34. *Proteobacteria* was the major bacterial phylum in all soil samples, followed by *Actinobacteria*, *Chloroflexi*, and *Acidobacteria*, accounting for a total of approximately 69% of the total abundance in each sample (Fig. 1a).

A total of 238 fungal OTUs were assigned to 21 fungal phyla: D contained 19 and H contained 19; *Ascomycota* was the major fungal phylum in all soil samples, followed by *Basidiomycota*, accounting for approximately 91% of the total abundance (Fig. 1b).

By comparing the α-diversities of D and H in terms of bacteria, a significant difference was observed between the OTU number, the Chao index (richness), and the Shannon index (diversity) of the samples; no significant difference was observed in the fungi (Table 1).

**Table 1:** Bacterial and fungal α-diversity index of different samples

Samples	OTUs (97%)	Chao index	Shannon index
Bacteria			
D	1143.44 ± 31.65a	3574.26 ± 81.95a	6.26 ± 0.04a
H	1474.67 ± 13.33b	4279.61 ± 171.97b	6.46 ± 0.04b
Fungi			
D	85.78 ± 1.69a	192.42 ± 7.68a	2.85 ± 0.04a
H	84.33 ± 1.45a	191.36 ± 9.25a	2.76 ± 0.04a

D = diseased soil, H = healthy soil. Values are expressed as the mean ± standard error. Means followed by the same letter for a given factor are not significantly different (P < 0.05; Tukey’s honest significant difference test)

**Fig. 2:**
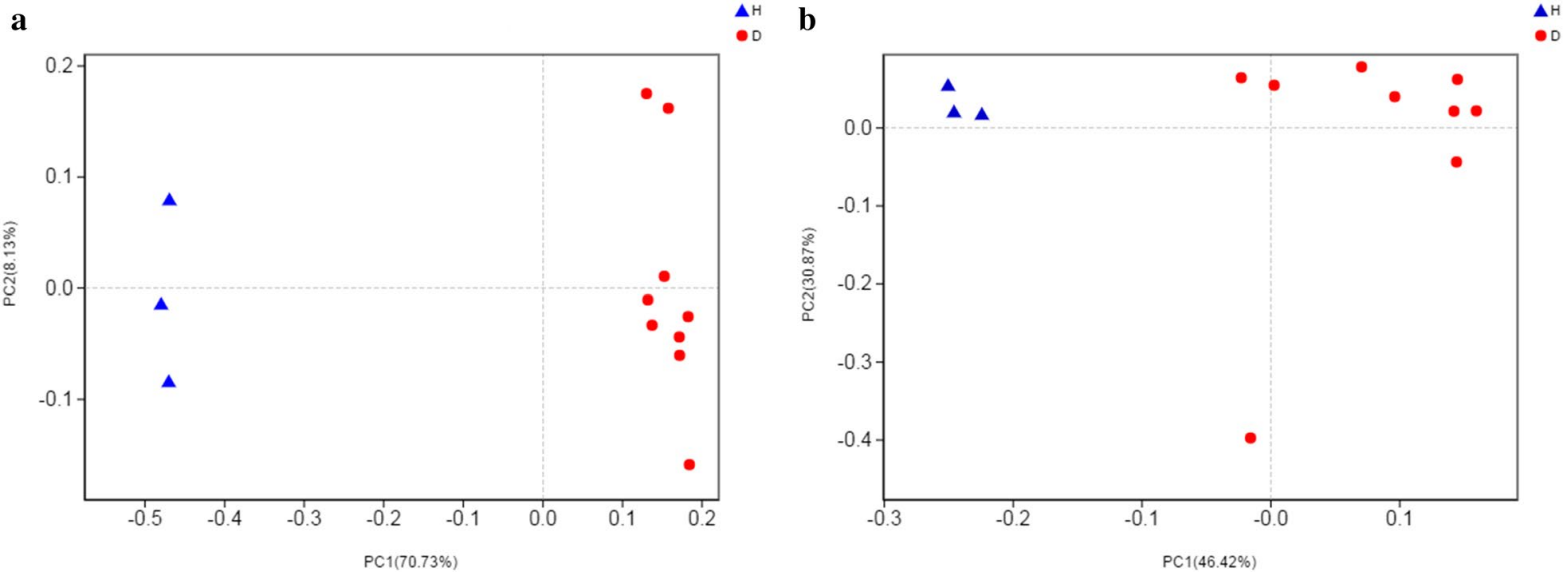
D and H β-diversity analyses: **a** bacterial PCoA analysis; **b** fungal PCoA analysis. XC1 ~ 9 are duplicates of D; HJ1 ~ 3 are duplicates of H

Statistical analysis of β-diversity involved the comparison of the composition of microbial communities. It was performed to determine the community treatment relationship between D and H. At the OTU level, PCoA was performed on each sample, and the distribution of D and H was significantly different regardless of bacteria or fungi (Fig. 2).

**Fig. 3:**
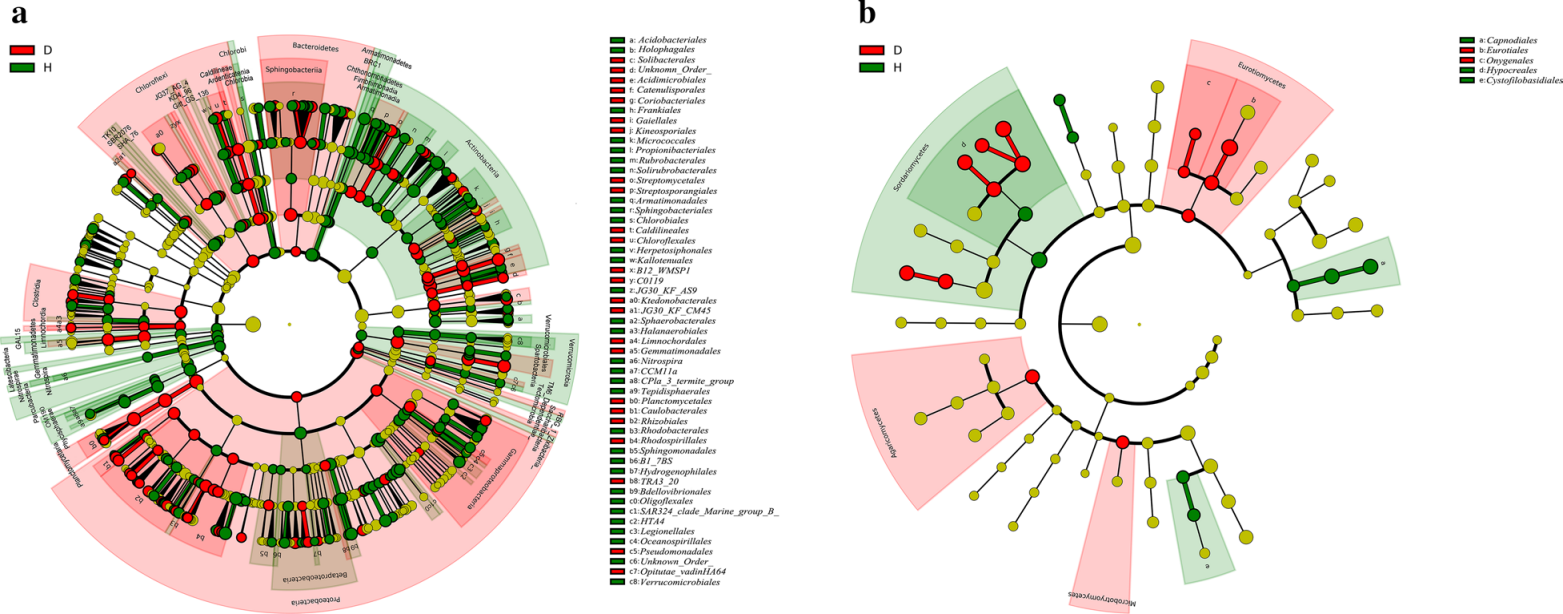
LEfSe cladogram of the aggregated groups of D and H. A range of bacterium (**a**) and fungus (**b**) taxa from phylum to genus level was associated with D (red) and H (green) (α = 0.05, LDA > 2.0, the size of circles is proportional to each taxon’s mean relative abundance). The yellow circles represent the absence of significantly different taxa

LEfSe was used to identify the dominant phylotypes responsible for the differences between D and H (Fig. 3). A total of 321 bacterial taxa were distinguished for the two groups: 117 for D and 204 for H; whereas 23 fungal taxa were distinguished for the two groups: 14 for D and 9 for H. At the genus level, 77 bacteria and 2 fungi were screened from H, whereas 43 bacteria and 4 fungi were screened from D (Additional file 2: Table S2 and Additional file 3: Table S3). The bacterium and fungus with the highest LDA value in H was *Nocardioides* (logarithmic LDA score = 3.67) and *Galactomyces* (logarithmic LDA score = 5.35), respectively (Additional file 2: Table S2 and Additional file 3: Table S3). The bacteria with the highest LDA value in D was *Chthoniobacter* (logarithmic LDA score = 4.28), and the fungi with the highest LDA value was *Claviceps* (logarithmic LDA score = 5.80) (Additional file 2: Table S2 and Additional file 3: Table S3). According to existing research reports, *Pseudomonas* was also found to differ in H and D at the genus level; it ranks 21st in H according to LDA value (logarithmic LDA score = 2.87). However, its relative abundance in H was 0.16%, whereas in D it was only 0.02% (Additional file 2: Table S2). The remaining differential microbial populations have not been found to have biocontrol functions in RKN.

### Changes in the microbial community in rhizosphere soil after treatment with biocontrol agents

Based on the results of previous studies, the genus known to have a biological control function and is present in the sequencing results of the present study was further investigated. The commonly used model strain *Pseudomonas*, *Pseudomonas fluorescens* (Pf), was selected from bacteria, while the control strain of *Bacillus subtilis* (Bs), which is common in the market, was used as control. In fungus, *Paecilomyces lilacinus* (Pl), which had parasitic effects on the female and eggs of RKN, was selected as a control. A blank control (P_D) was also set.

Through high-throughput sequencing analyses of 16S rDNA and 18S rDNA, 457,260 valid bacterial sequences and 447,312 effective fungal sequences were read from 12 field samples and divided into 5094 bacterial OTUs and 293 fungal OTUs.

**Fig. 4:**
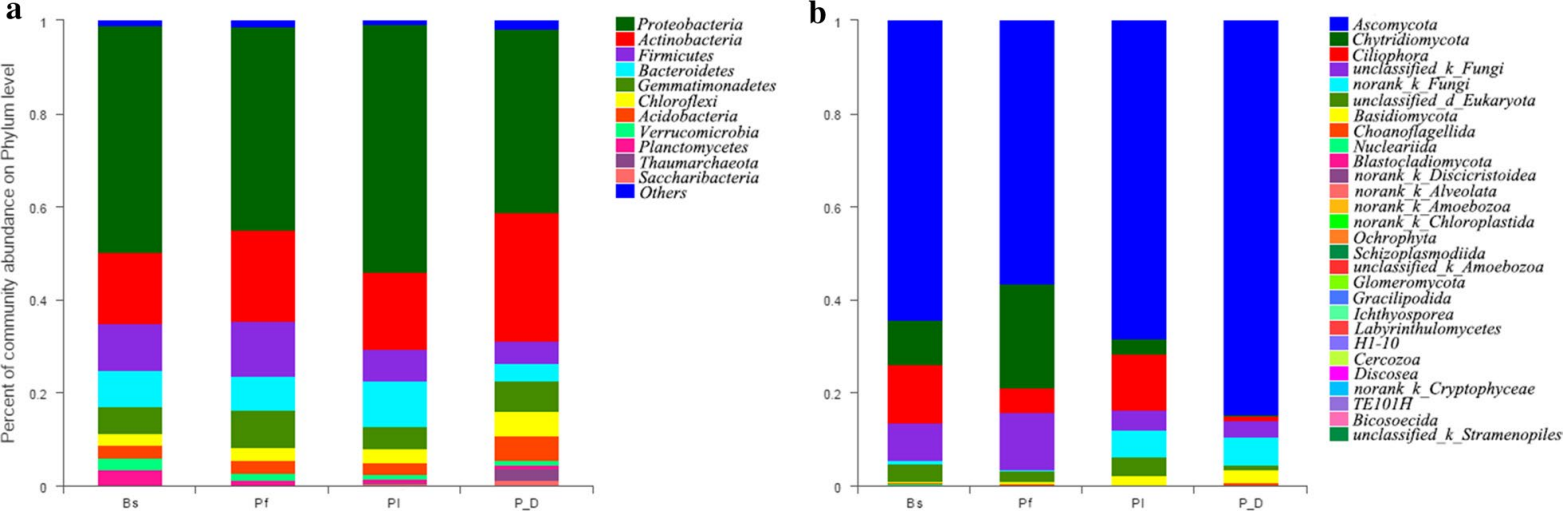
Bacterial (**a**) and fungal (**b**) community composition after treatment with biocontrol agents: *Bacillus subtilis* (Bs), *Pseudomonas fluorescens* (Pf), and *Paecilomyces lilacinus* (Pl); the untreated diseased soil (P_D) was set as control

A total of 5094 bacterial OTUs were assigned to 39 different phyla: Bs contains 28, Pf contains 33, Pl contains 31, and P_D contains 36. *Proteobacteria* was the major bacterial phylum in all soil samples, followed by *Actinobacteria* and *Firmicutes*, accounting for a total of approximately 66% of the total abundance in each sample (Fig. 4a).

A total of 293 fungal OTUs were assigned to 28 fungal phyla: Bs contains 24, Pf contains 21, Pl contains 23, and P_D contains 26. *Ascomycota* was the major fungal phylum in all soil samples, followed by *Chytridiomycota*, accounting for approximately 75% of the total abundance in each sample (Fig. 4b).

By comparison of the α-diversity of different treatments in terms of bacteria, OTUs and Chao indices (richness) of Bs and Pf were found to be significantly different compared with those of Pl and P_D, whereas the Shannon indices (diversity) of all three treatments were found to be significantly different from that of P_D. After treatment, the richness and diversity decreased. In terms of fungi, Bs and Pf treatments showed significant differences in OTU and Chao index (richness) compared with Pl or P_D; Bs treatment exhibited significantly different values compared with the other three Shannon indices (diversity). Overall, post-treatment diversity and richness exhibited a decreasing trend (Table 2).

**Table 2:** Bacterial and fungal α-diversity indices of different samples after treatment with biocontrol agents

Samples	OTUs (97%)	Chao index	Shannon index
Bacteria			
Bs	2009.33 ± 104.95a	2898.44 ± 220.29a	5.65 ± 0.11a
Pf	2105.67 ± 43.46a	2968.13 ± 45.00a	5.81 ± 0.05a
Pl	2437.33 ± 81.72b	3660.34 ± 238.42b	5.87 ± 0.08a
P_D	2549.67 ± 99.63b	3731.33 ± 135.10b	6.31 ± 0.08b
Fungi			
Bs	137.67 ± 3.71a	168.49 ± 3.88a	2.08 ± 0.17a
Pf	142.00 ± 2.08a	164.46 ± 2.07a	2.62 ± 0.05b
Pl	163.33 ± 5.24b	203.70 ± 7.03b	3.11 ± 0.13b
P_D	198.33 ± 7.84c	240.06 ± 11.47c	2.77 ± 0.04b

**Fig. 5:**
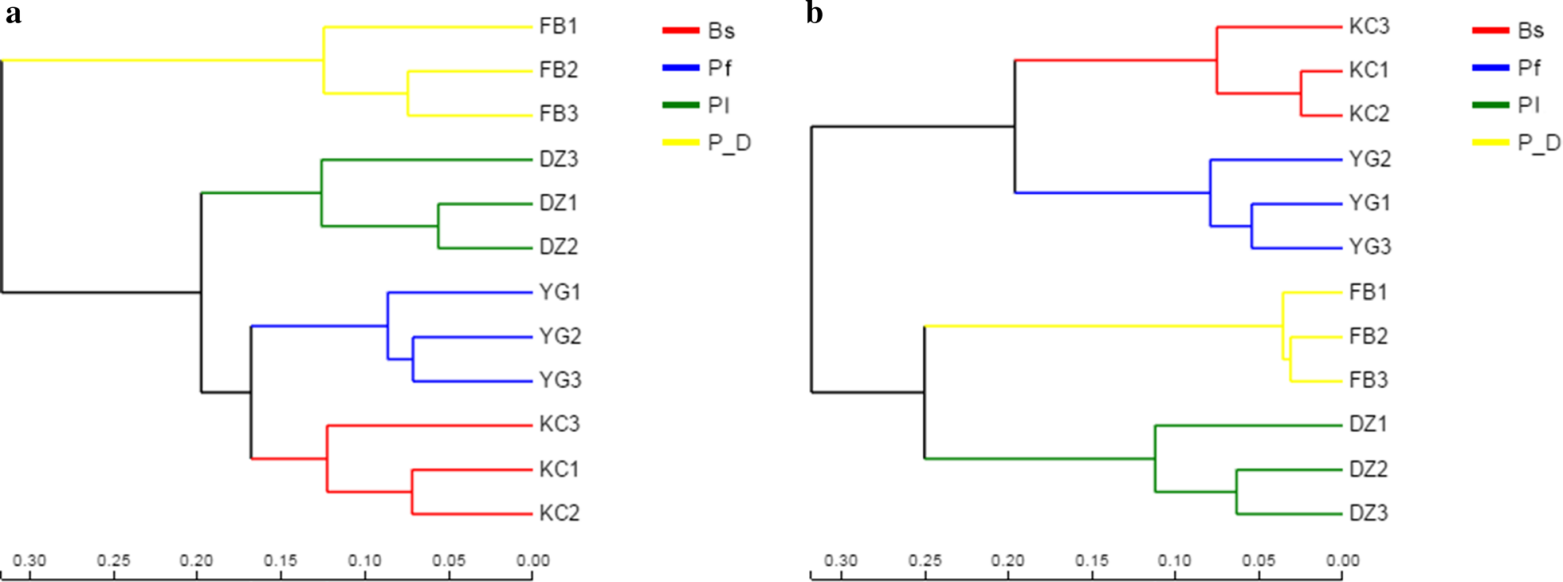
β-diversity analysis after treatment: **a** bacterial hierarchical clustering analysis; **b** fungal hierarchical clustering analysis. Numbers 1 to 3 refer to the duplicates of each treatment

At the OTU level, hierarchical clustering analysis was performed on each sample. Clustering differences were observed among the samples treated, regardless of bacteria or fungi (Fig. 5).

LEfSe analysis was performed to discriminate the main microbial differences between the control soil P_D and the treated soils Bs, Pf, and Pl (Additional file 4: Fig. S1). In terms of bacteria, genus was selected for analysis. Bs and P_D were compared: 208 data were divided into two groups, Bs accounted for 134 (Additional file 4: Fig. S1a, Additional file 5: Table S4); Pf was compared with P_D: 218 data were divided into two groups, Pf accounted for 136 (Additional file 4: Fig. S1b, Additional file 6: Table S5); and Pl and P_D were compared: 192 data were divided into two groups, Pl accounted for 157 (Additional file 4: Fig. S1c, Additional file 7: Table S6). For fungi, the genus level was selected for analysis, Bs and P_D were compared, 13 were different data, Bs accounted for 5 (Additional file 4: Fig. S1d, Additional file 8: Table S7); Pf was compared with P_D, 8 difference data, Pf accounted for 8 (Additional file 4: Fig. S1e, Additional file 9: Table S8); Pl and P_D were compared, 7 difference data, Pl accounted for 1 (Additional file 4: Fig. S1f, Additional file 10: Table S9).

### Comparison of changes in different microbial populations before and after treatment

**Fig. 6:**
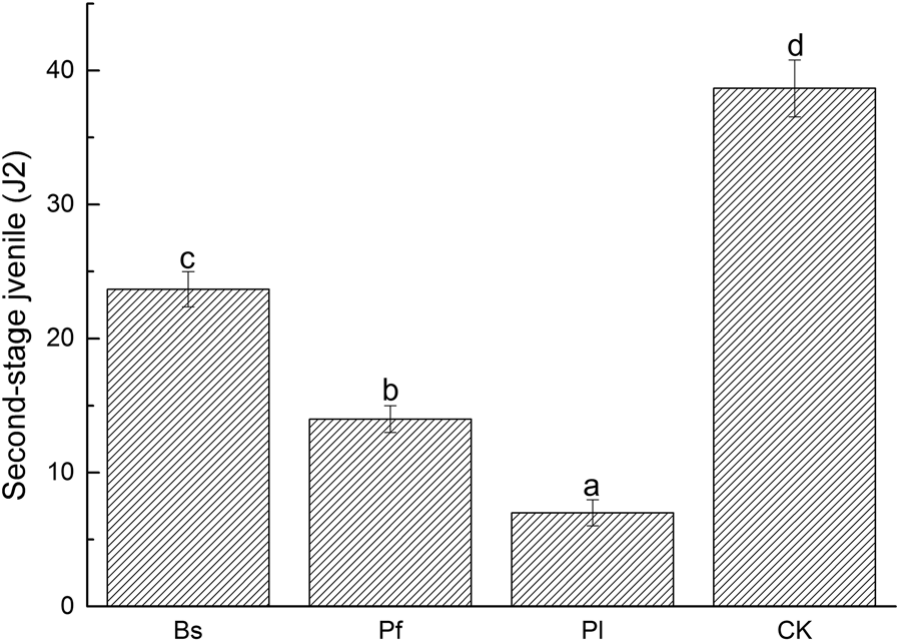
Population of the second-stage juvenile of RKN (J2) per treatment (100 g of rhizosphere soil)

Rhizosphere soil (100 g) was collected from each treatment to determine the second-stage juvenile population of RKN (J2). The Pl treatment content was the lowest, with an average of 7; Pf contained 14; Bs had an average of 23.66; and that of the untreated diseased soil was 38.67. A significant difference was observed between the treatments and the control (Fig. 6).

**Fig. 7:**
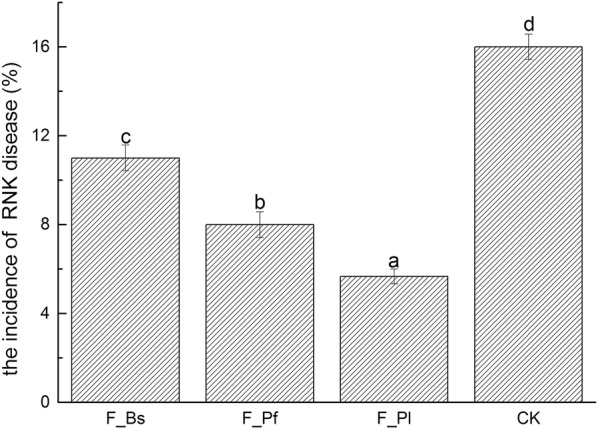
RKN incidence in the field

Field trials with different treatments showed significant differences in the number of RKN incidence (Fig. 7). In general, all three treatments reduced the incidence of tobacco RKN infestation. Compared with the control, F_Pl, F_Pf, and F_Bs reduced the incidence rates by 64.58%, 50.00%, and 31.25%, respectively.

Pearson correlation analysis revealed that significant correlations exist between the 16 genera and J2 at the bacterial genus level (11 negative correlations, 5 positive correlations) (Table 3). The relative abundance and difference in H and D were also calculated as shown in Table 3. No difference was observed in the relative abundance of *Pontibacter* and *Massilia* in H and D, whereas the 14 other genera exist.

**Table 3:** Correlation between J2 and bacterial community at the genus level, and relative abundance (%) in H and D

Genus	H	D	r	P
*Microvirga*	0.17 ± 0.01a	0.02 ± 0.00b	− 0.8588	0.0003
*Pedobacter*	0.15 ± 0.01a	0.01 ± 0.00b	− 0.8514	0.0004
*Diaphorobacter*	0.35 ± 0.04a	0.08 ± 0.01b	− 0.8249	0.0010
*Pontibacter*	0.00 ± 0.00a	0.00 ± 0.00a	− 0.8225	0.0010
*Sphingomonas*	1.99 ± 0.16a	1.28 ± 0.10b	0.7853	0.0025
*Massilia*	0.13 ± 0.02a	0.13 ± 0.01a	− 0.7843	0.0025
*Ramlibacter*	1.82 ± 0.02a	0.44 ± 0.06b	− 0.7739	0.0031
*Lysobacter*	0.68 ± 0.04a	0.10 ± 0.01b	− 0.7695	0.0034
*Bradyrhizobium*	1.43 ± 0.06a	2.23 ± 0.15b	0.7367	0.0063
*Bryobacter*	0.82 ± 0.03a	3.02 ± 0.27b	0.7338	0.0066
*Pseudomonas*	2.50 ± 0.11a	0.49 ± 0.05b	− 0.7319	0.0068
*Pseudolabrys*	0.37 ± 0.03a	1.21 ± 0.11b	0.7155	0.0089
*Rhodanobacter*	0.14 ± 0.02a	0.60 ± 0.05b	− 0.6760	0.0158
*Adhaeribacter*	0.05 ± 0.01a	0.00 ± 0.00b	− 0.6587	0.0198
*Shinella*	0.23 ± 0.02a	0.01 ± 0.00b	− 0.6412	0.0246
*Variibacter*	0.73 ± 0.03a	1.73 ± 0.12b	0.6128	0.0341

Pearson correlation revealed that no significant correlations exist between the nine genera and J2 at the fungal genus level (two negative correlations, seven positive correlations) (Table 4). The relative abundance and difference in H and D were also calculated as shown in Table 4. No difference was observed in the relative abundance of *Pseudallescheria*, *Nuclearia*, *Rhizoctonia* and *Mrakia* in H and D, whereas the 5 other genera exist.

**Table 4:** Correlation between J2 and the fungal community at genus level, and relative abundance (%) in H and D

Genus	H	D	r	P
*Cladosporium*	5.47 ± 0.33a	0.96 ± 0.04b	0.8248	0.0010
*Cryptococcus*	0.57 ± 0.03a	0.29 ± 0.02b	0.8245	0.0010
*Coniochaeta*	1.30 ± 0.03a	2.35 ± 0.09b	0.7698	0.0034
*Pseudallescheria*	0.52 ± 0.09a	0.28 ± 0.02a	0.7579	0.0042
*Pyxidiophora*	0.36 ± 0.02a	0.20 ± 0.03b	0.7422	0.0057
*Nuclearia*	0.00 ± 0.00a	0.00 ± 0.00a	− 0.6195	0.0317
*Rhizoctonia*	0.01 ± 0.00a	1.75 ± 0.10a	− 0.6159	0.0330
*Metarhizium*	0.27 ± 0.02a	7.52 ± 0.11b	0.6157	0.0330
*Mrakia*	0.02 ± 0.00a	0.07 ± 0.00a	0.6048	0.0372

**Fig. 8:**
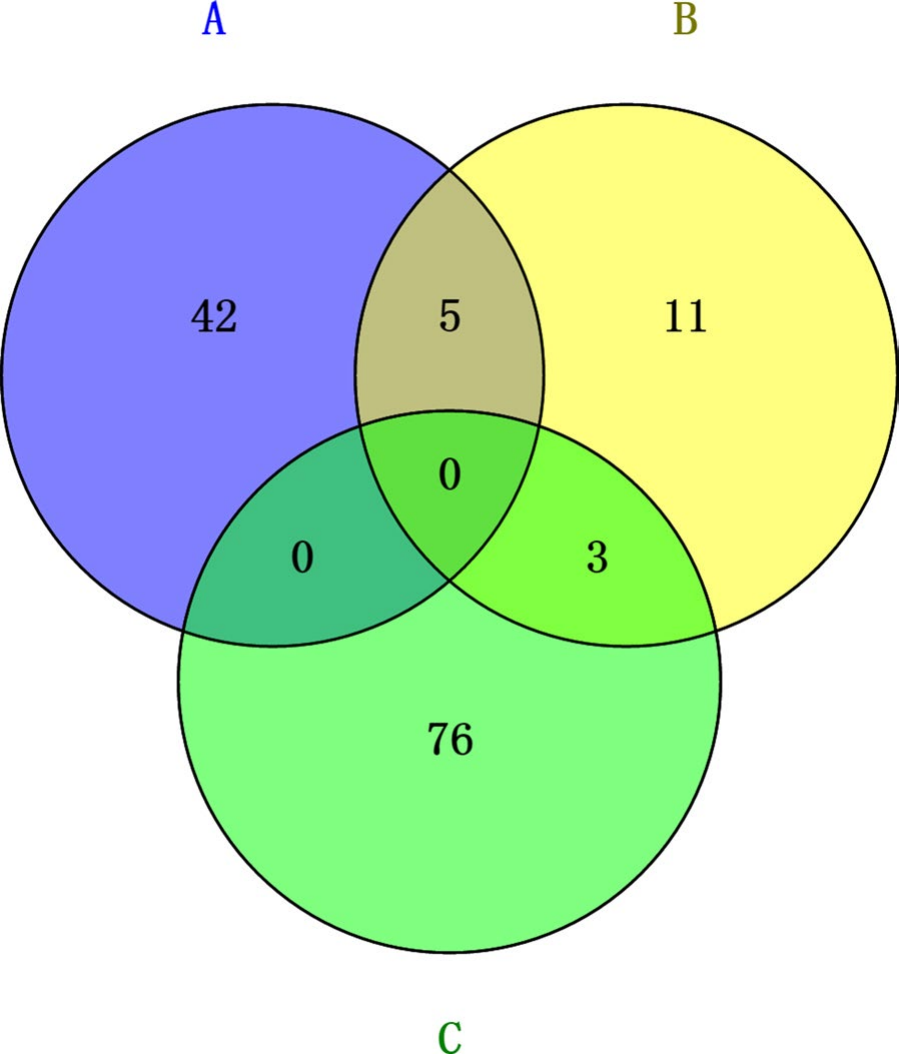
Differential microbial comparison. A = Differential microorganisms in diseased soil. B = Differential microorganisms associated with J2 after treatment. C = Differential microorganisms in healthy soil

From the Venn diagrams (Fig. 8), we found that in terms of bacteria, in the genus that was positively correlated with the number of J2, *Sphingomonas* was found in H and *Bryobacter* and *Variibacter* were found in D; in the genus that was negatively correlated with the number of J2, *Pseudomonas* was found in H and *Adhaeribacter* was found in D. In terms of fungi, we found that *Cladosporium* was positively correlated with J2 in H, and *Coniochaeta* and *Metarhizium* were in D.

## Discussion

Biocontrol agents can significantly affect soil microbial community structure, improve soil microecological environment, and inhibit disease occurrence (Zhang et al. 2013; Xiong et al. 2015). According to research reports, Bs, Pf, and Pl are all biocontrol agents that have a controlling effect on RKN disease. Among them, Bs and Pf are reported to have a control effect or have inhibitory effects on nematodes, and Pl is an egg parasitic fungus (Araújo and Marchesi. 2009; Siddiqui et al. 2006; Nagesh et al. 2013). In our study, pot experiments confirmed that biocontrol agents, Bs, Pf, and Pl, were effective in reducing the number of second-stage juvenile of RKN. Meanwhile, field trials showed that the incidence rates were reduced to 64.58%, 50.00%, and 31.25% (Figs. 6 and 7). This finding is similar to the conclusion by Seo et al. that a control effect on RKN disease is achieved by using microbial agents (Seo et al. 2012).

The change in soil microbial community is the most important biological factor that affects the occurrence of soil-borne diseases (Kloeppe et al. 1999; Larkin 2008). In previous studies, the occurrence of RKN disease was found to be closely related to the interaction between soil microbial communities (Echeverrigaray et al. 2010). Our study found that the incidence of this disease was determined by comparison with healthy soil and the difference between the Chao and Shannon indices of the bacteria. No difference was observed in all aspects of the fungi (Table 1). This finding may be related to the fact that the abundance and species of the fungi in soil are extremely less than that of bacteria (Yang et al. 2018a, b). By treatment with biocontrol agents, the overall diversity and richness decreased after treatment (Table 2). This finding may be due to the fact that when a large number of microorganisms are added to the soil, the indigenous microbial community structure changes and various characteristics are exhibited (Zhang et al. 2019).

To determine the difference between the diseased and the healthy soil microbes, as well as the difference in microbial abundance after treatment, we used LEfSe analysis to compare the phylum and the genus levels, which means that the indicator is sufficiently distinguishable in the sample. At the phylum level, the overall microbial species composition is not considerably different, because the structure of the soil microbial community is not very different (Greening et al. 2015). At the genus level, we found 81 bacteria and 2 fungi in the healthy soil and 47 bacteria 4 fungi in the diseased soil (Fig. 3, Additional file 2: Tables S2 and Additional file 3: Table S3). After treatment, at the genus level, compared with the control in terms of bacteria, Bs has 134, Pf has 136, and Pl has 157; in terms of fungi, Bs has 5, Pf has 8, and Pl has only 1 (Additional file 4: Fig. S1, Additional file 5: Table S4, Additional file 6: Table S5, Additional file 7: Table S6, Additional file 8: Table S7, Additional file 9: Table S8, Additional file 10: Table S9). The number of different species after treatment increased significantly. Microbial agents were reported to improve the differences in soil microbial abundance, affect soil microbial community structure, and further alter soil microecological functions (Buyer et al. 2011; Parsa et al. 2018).

The abundance of pathogens is closely related to the occurrence of diseases, and the second-stage juvenile population in RKN in the soil can affect the degree of infection (Bartlem et al. 2014). We studied the Pearson correlation between the number of second-stage juvenile after treatment and the microbes in the soil and found that 16 genera bacteria and 9 genera fungi were related (Tables 3 and 4). Then, we delved into the differences in the abundance in the diseased and the healthy soils. We found that only 14 genera bacteria and 5 genera fungi exhibited significant differences, and these microorganisms have positive or negative correlation with the number of second-stage juvenile. At this time, we carried out further research and compared it with the Venn diagram of the differential microorganisms in the diseased and the healthy soils we found earlier, and found the microbial part with intersection (Fig. 8). According to our research, the bacteria *Pseudomonas* was negatively correlated with soil health. At the same time, we found that the bacteria *Bryobacter* and *Variibacter*, as well as fungi *Coniochaeta* and *Metarhizium*, were positively related to soil health. According to related reports, these five types of microorganisms are key microbial groups that affect the occurrence of RKN disease (Liu et al. 2016, Cao et al. 2015).

From the perspective of the individual biological functions of these types of microorganisms, as a group of high-abundance bacteria in the soil, *Pseudomonas* plays an important role in plant disease inhibition and plant growth (Patten and Glick. 2002, Abramovitch et al. 2003). Moreover, *Pseudomonas* is a Gram–negative bacterium belonging to *Pseudomonadaceae*; it is beneficial for inhibiting soil-borne diseases and providing a healthy soil environment to promote root growth (Ma et al. 2018). At present, numerous reports have been made on the application of *Pseudomonas*, including agricultural biological control, plant growth regulation, environmental protection, and medical development (Mandelbaum et al. 1995). Among them, the research on biological control and plant growth is the most thorough and widely used. The main targets of biological control are phytopathogenic fungi, such as *Fusarium*, *Verticillium dahliae*, and *Anthrax* (Aparna et al. 2012, Xu et al. 2017). Studies have reported that *Pseudomonas* also has a good inhibitory effect on RKNs (Siddiqui et al. 2002). Notably, the analysis of soil microorganisms related to disease occurrence should target one or several specific types and be directed to the interaction among soil microbial communities (Shen et al. 2018).

At present, the biological control of soil-borne diseases is primarily concentrated on beneficial microorganisms that can be cultured; the disease resistance mechanism has been reported in detail, but the resistance and resilience of the microbial communities in the soil communities have not been reported (Deshwa et al. 2003, Mazzola and Freilich 2016, Topp et al. 2017). Based on the 16S rRNA and 18S rRNA gene sequencing studies, we have broadly expanded our understanding on soil microbial diversity and began to reveal more parts of soil microbial communities that are not cultured. However, we cannot fully link all soil microbiome to RKN disease; hence, identifying several important microorganisms that have great potential for disease occurrence is highly feasible. In conclusion, the addition of exogenous beneficial microorganisms in the soil has a certain inhibitory effect on RKN disease, which may be related to the direct or indirect changes in some microorganisms, further affecting the structure and function of the microbial community in the rhizosphere soil. This study only discussed the changes in soil microbes and their relationship with the occurrence of a small number of biocontrol agents. The five genera with abundance and correlation were indicative of the great potential of tobacco RKN disease. Future research must be conducted to investigate these genera and prevent disease by controlling their specificity.

## Supplementary information

**Additional file 1: Table S1.** The basic physical and chemical properties of each soil sample.

**Additional file 2: Table S2.** Diseased and healthy soil differential bacteria.

**Additional file 3: Table S3.** Diseased and healthy soil differential fungi.

**Additional file 4: Figure S1.** LEfSe analysis between the control soil P_D and the treated soils Bs, Pf, and Pl.

**Additional file 5: Table S4.** Difference bacteria between Bs and P_D.

**Additional file 6: Table S5.** Difference bacteria between Pf and P_D.

**Additional file 7: Table S6.** Difference bacteria between Pl and P_D.

**Additional file 8: Table S7.** Difference fungi between Bs and P_D.

**Additional file 9: Table S8.** Difference fungi between Pf and P_D.

**Additional file 10: Table S9.** Difference fungi between Pl and P_D.

## Data Availability

The dataset supporting the conclusions of this article is included within the article and its additional files.

## Abbreviations

RKN: Root-knot nematode
FAO: Food and Agriculture Organization of the United Nations
rRNA: Ribosomal ribonucleic acid
D: Diseased-soil
H: Healthy-soil
Bs: *Bacillus subtilis*
Pf: *Pseudomonas fluorescens*
Pl: *Paecilomyces lilacinus*
P_D: Untreated diseased soil
J2: The population of the second-stage juveniles of RKN
CK: Without any treatment
F_Bs: 100 billion live spores/g *Bacillus subtilis*
F_Pf: 300 billion live spores/g *Pseudomonas fluorescens*
F_Pl: 5 billion live spores/g *Paecilomyces lilacin*
DNA: Deoxyribonucleic acid
PCR: Polymerase chain reaction
PCoA: Principal coordinates analysis
LEfSe: Least discriminant analysis effect size
LDA: Linear discriminant analysis
OTU: Operational taxonomic units
